# First-in-human prospective pilot study of a bipolar dual-center total wrist arthroplasty: early mechanical stability and clinical outcomes

**DOI:** 10.1007/s00402-026-06493-z

**Published:** 2026-10-22

**Authors:** Raphael Israeli, Lauren Gorelick, Gil Gannot, Amir Oron

**Affiliations:** 1 Department of Orthopedic Surgery, Kaplan Medical Center, Rehovot, Israel; 2 Clalit Health Services, Tel Aviv, Israel; 3 Faculty of Medicine, Hebrew University of Jerusalem, Jerusalem, Israel

**Keywords:** Total wrist arthroplasty, Wrist replacement, Wrist biomechanics, SLAC wrist, SNAC wrist, Implant stability, Prospective pilot study

## Abstract

**Purpose:**

Total wrist arthroplasty (TWA) is limited by implant loosening, instability, and non-physiological load transfer, partly because most contemporary implants rely on a single center of rotation and do not preserve native wrist kinematics. This first-in-human pilot study evaluated the feasibility, early mechanical stability, and clinical outcomes of a novel bipolar dual-center TWA system designed to preserve radiocarpal and midcarpal motion.

**Methods:**

This prospective single-center pilot case series included five consecutive patients with end-stage wrist arthritis treated with a bipolar dual-center TWA implant using cement fixation, as per the device’s intended use. The outcome measures included pain assessed using the pain subscale of the Patient-Rated Wrist Evaluation (PRWE) questionnaire (normalized to a 0–10 scale), total PRWE score, DASH score, range of motion, grip strength, radiographic implant stability, complications, and reoperation. Clinical outcomes were assessed at the most recent follow-up visit.

**Results:**

The mean follow-up was 19.2 months (range, 12–24 months), with 24-month data available for three patients. Implant survivorship was 100%, with no loosening, migration, dislocation, or implant revision observed on radiographs. One patient experienced a traumatic postoperative fall without loss of implant fixation. The mean PRWE-derived pain score improved from 9.0 to 3.0, and the mean PRWE total improved from 84.6 to 23.2 at the final follow-up. The mean DASH score improved from 79.0 to 29.5, with substantial inter-patient variability (final range, 0–91.7). All five patients met the predefined responder criterion of ΔPRWE ≥ 20 combined with radiographic stability. Three patients with positive preoperative signs of carpal tunnel syndrome subsequently underwent carpal tunnel release 4–8 months postoperatively, with symptomatic improvement in all patients. One intraoperative carpal fracture was managed without component revision surgery. The mean wrist flexion improved from 13° to 37°, and extension improved from 11° to 32°.

**Conclusions:**

This first-in-human pilot study demonstrated the technical feasibility and early mechanical stability of the bipolar dual-center TWA concept. All patients met the predefined wrist-specific responder criteria with stable mechanical implant fixation. These findings support further investigation of the dual-center concept in larger studies with longer follow-up periods.

## Introduction

Total wrist arthroplasty (TWA) is a challenging surgical option for treating advanced wrist arthritis. Although modern implant designs have improved compared to those of earlier generations, long-term outcomes are limited by implant loosening, instability, mechanical failure, and inconsistent functional restoration [1, 2]. Consequently, many surgeons still consider total wrist fusion to be a more predictable option for patients with advanced degenerative wrist disease [3].

Unlike simple hinge joints, the native wrist is a highly complex biomechanical system composed of multiple articulations that function through coordinated radiocarpal and midcarpal motion. Physiological wrist kinematics depend on a balanced interaction between these articulations, allowing smooth multiplanar motion and controlled load transmission across the carpus [4]. Disruption of these relationships may alter force distribution patterns and increase mechanical stress at the implant–bone interface [5, 6].

Currently, most TWA systems rely on a single center of rotation and frequently require partial carpal fusion or substantial carpal bone resections. Although these designs may provide acceptable pain relief, they do not fully reproduce native wrist kinematics, particularly with respect to the preservation of midcarpal motion [5, 7, 8]. Consequently, abnormal load transfer and altered carpal mechanics may contribute to implant loosening and long-term mechanical failure [9, 10].

To address these limitations, a novel bipolar dual-center total wrist arthroplasty system (WristArt^™^; Fibioseq Medical Ltd., Israel) was developed. This implant is designed to preserve both the radiocarpal and midcarpal centers of rotation while avoiding unnecessary carpal fusion and minimizing bone resections. By maintaining carpal articulation and allowing motion to occur through two coordinated rotational centers, the design aims to better replicate native wrist biomechanics [11, 12], while reducing non-physiological load transfer [5, 13]. It was hypothesized that this dual-center configuration would promote more physiological kinematics, mitigate abnormal mechanical stress, and provide early mechanical stability without compromising the motion [5, 14, 15].

This pilot study reports the first-in-human cases evaluating the safety, feasibility, implant stability, and early clinical outcomes of a novel bipolar dual-center wrist arthroplasty system in five consecutive patients.

## Methods

### Study design

This prospective single-center series evaluated a novel bipolar dual-center total wrist arthroplasty (WristArt^™^; Fibioseq Medical Ltd., Israel). The study included the first five consecutive patients who underwent device implantation at our institution between January and September 2024. Institutional Review Board approval was obtained prior to study initiation, and all patients provided written informed consent for participation and publication of this study. Clinical and radiographic data were prospectively collected at each follow-up visit.

### Patient selection

Five consecutive patients with symptomatic end-stage wrist arthritis refractory to nonoperative treatment were included in this study. The indications for surgery were post-traumatic osteoarthritis, scapholunate advanced collapse (SLAC) wrist, scaphoid nonunion advanced collapse (SNAC) wrist, and severe primary wrist osteoarthritis.

The implant is intended for patients with end-stage wrist arthritis of various etiologies, including post-traumatic osteoarthritis, primary osteoarthritis, SLAC and SNAC wrists, and inflammatory arthropathies, including rheumatoid arthritis (RA). Patient selection was based on clinical symptoms, functional limitations, radiographic evidence of advanced degenerative disease, and surgeon assessments. Patients with active infections or severe bone loss that precluded fixation were not considered for implantation.

Furthermore, no patients in this cohort had a history of Complex Regional Pain Syndrome (CRPS) or other pre-existing chronic pain syndromes, and none of the patients with SNAC or SLAC wrist had undergone prior wrist surgeries. Additionally, patients with inadequate bone stock, specifically those with ≥ 50% capitate destruction or poor-quality radial bone, were excluded from implantation.

### Surgical procedure

All procedures were performed by a single senior surgeon using a dorsal wrist approach between the third and fourth extensor compartments. The extensor pollicis longus tendon was mobilized, and the dorsal capsular flap was elevated. The lunate and proximal scaphoid bones were excised to create a carpal preparation bed. Under fluoroscopic guidance, a central guidewire was inserted through the capitate, followed by sequential preparation of the capitate and distal radius using dedicated instruments and reamers.

**Fig. 1:**
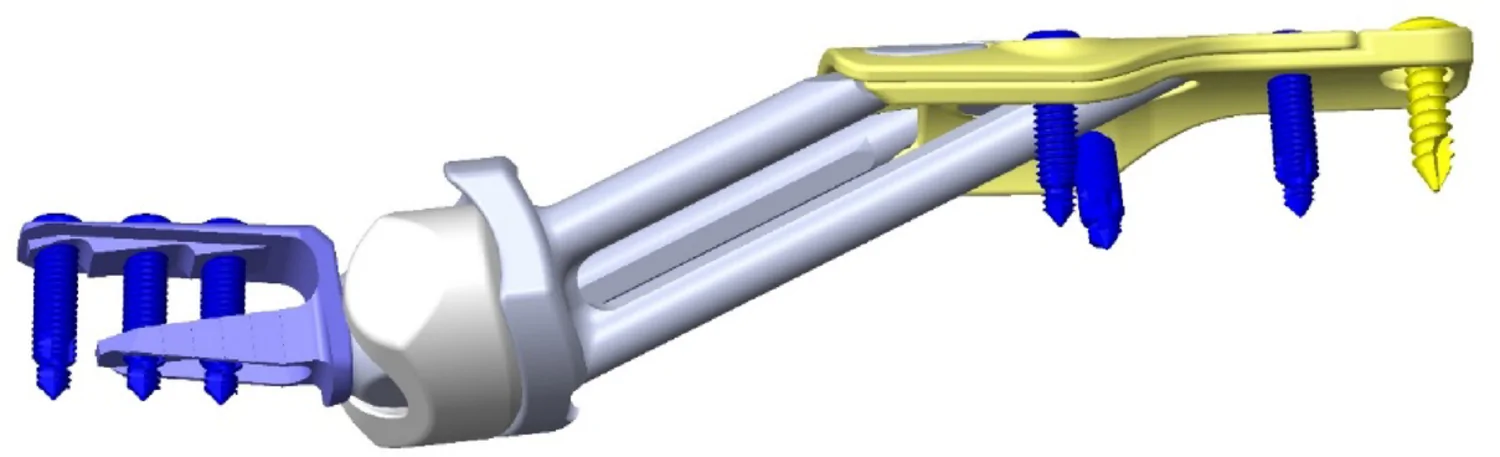
Schematic overview of the WristArt^™^ bipolar dual-center total wrist arthroplasty implant. The implant consists of a radial component, a carpal component, a neck component, and an articulating joint component. The radial component incorporates an intramedullary stem and a dorsal fixation plate. The implant is universal, suitable for both right and left wrists, and utilizes a one-size-fits-all design, while the neck component is available in four sizes to adjust the tension level in the joint. The design reproduces a dual-mobility kinematic concept through two centers of rotation: a midcarpal ball joint within the carpal component and a radiocarpal articulation between the elliptical surface and the radial component

The WristArt^™^ implant consists of cobalt–chromium–molybdenum (CoCrMo) and titanium components (Fig. 1). Articulation occurs between a highly polished CoCrMo surface and a highly cross-linked, vitamin E–stabilized ultra-high molecular weight polyethylene (UHMWPE) insert.

The implant was designed for cemented fixation in accordance with its intended regulatory pathway; therefore, cement fixation was used in all cases. A medium-viscosity polymethyl methacrylate (PMMA) bone cement (SMARTSET^™^ MV ENDURANCE^™^, DePuy Synthes, Johnson & Johnson MedTech) was prepared using a vacuum-mixing system. After confirming a satisfactory implant position and alignment, approximately 0.5 mL of bone cement was applied to the radial and carpal bone–implant interfaces. Cement was used to fill the residual gaps between the radial component and the distal radius, and between the carpal component and the proximal surface of the capitate. This technique provides stable primary fixation while minimizing cement extrusion. Because only a thin cement mantle was used, the cement may not be visible on standard postoperative radiographs.

A dedicated alignment guide was used to introduce the radial stem through the dorsal cortex into the radial intramedullary canal, followed by the fixation of a dorsal plate over the intramedullary component. Trial implantation was performed in all cases to assess implant alignment and passive wrist motion prior to the final implantation. The joint capsule and surrounding soft tissues at the surgical site were meticulously closed. A bulky dressing was applied after the procedure.

### Postoperative care

Postoperative rehabilitation adhered to a standardized, study-specific protocol that delineated the initial assessment, duration, and frequency of occupational therapy. Although the protocol established explicit principles and milestones, it permitted individualized progression and case-specific modifications based on the operating surgeon’s discretion, soft-tissue conditions, and patient tolerance. During the first two postoperative weeks, the protocol mandated wrist immobilization in a splint, emphasizing edema control, wound care, pain management, and active and passive exercises of the shoulder, elbow, fingers, thumb, and forearm (pronation and supination). In this initial phase, wrist loading, resistance exercises, and weight-bearing activities were strictly prohibited. Commencing two weeks postoperatively, active and active-assisted wrist range-of-motion exercises were introduced, with a dart-throwing motion incorporated from postoperative day 21. Between weeks 7 and 12, passive wrist mobilization and progressive strengthening were initiated, while axial loading remained restricted; patients were advised to avoid lifting more than 4 kg during this period and no more than 5 kg thereafter.

### Outcome assessment

Patients were prospectively followed up at approximately 1, 3, 6, 12, and 24 months postoperatively. Extended 24-month follow-up data were available for three patients (KA-002-A, KA-004-B, and KA-006-D), whereas two patients (KA-005-C and KA-007-E) completed the 12-month follow-up at the time of the analysis. Clinical outcomes were analyzed using the latest follow-up data available for each patient.

Outcome measures included pain assessed using the pain subscale of the Patient-Rated Wrist Evaluation (PRWE) questionnaire. The PRWE pain subscale consists of five items, each scored from 0 (no pain) to 10 (worst pain), yielding a summed score of 0–50. For consistency with the conventional 0–10 pain scale familiar in clinical practice, the summed score was divided by five to yield a normalized average pain score on a 0–10 scale. This transformation preserves the underlying psychometric properties of the PRWE pain subscale while providing values comparable to those of traditional pain assessment instruments. Wrist-specific function was assessed using the PRWE total score and global upper-extremity disability using the Disabilities of the Arm, Shoulder, and Hand (DASH) questionnaire. The wrist range of motion was measured using a standard goniometric technique with the forearm maintained in a neutral rotation. Grip strength was measured in kilograms using three consecutive trials for each hand, and mean values were recorded.

Postoperative radiographs (posteroanterior and lateral views) were obtained at each follow-up visit and assessed for implant alignment, fixation, loosening (defined as radiolucent lines ≥ 2 mm at the bone–implant interface), subsidence, migration, dislocation, and component-related mechanical failure. The operating surgeon reviewed radiographs at each visit.

Complications, secondary procedures, and reoperations were prospectively documented.

### Definition of clinical response

A hierarchical responder definition was used, combining patient-reported wrist-specific function and objective mechanical stability. Patients were classified as responders if both criteria were met: (1) ΔPRWE ≥ 20 from baseline to the latest follow-up, exceeding the MCID of 11.5 reported by Walenkamp et al. [16] and falling within the MCID range of 17–24 reported in a recent meta-analysis [17], and (2) absence of radiographic signs of implant loosening. The DASH score was used as a secondary global surveillance measure; in cases of discordance between metrics, the responder status was determined based on the primary wrist-specific data.

### Statistical analysis

Given the exploratory nature and small sample size of this study, the data analysis was descriptive in nature. Continuous variables are presented as mean values, standard deviations, ranges, and individual patient trajectories, as appropriate. No formal hypothesis testing was performed because of the exploratory nature and the limited sample size of the cohort.

## Results

### Patient cohort and baseline characteristics

Five consecutive male patients underwent WristArt^™^ bipolar dual-center TWA. The mean age at the time of surgery was 67.0 years. The surgical indications were post-traumatic osteoarthritis (*n* = 1), scapholunate advanced collapse (SLAC) wrist (*n* = 2), scaphoid nonunion advanced collapse (SNAC) wrist (*n* = 1), and severe primary wrist osteoarthritis (*n* = 1). The operated sides included three right and two left wrists. The baseline demographic, diagnostic, and preoperative outcome characteristics are summarized in Table 1.

**Table 1:** Baseline patient characteristics and preoperative outcome measures

Patient ID	Age, years	Sex	Diagnosis	Side	Follow-up, months	PRWE	DASH	Pain*
KA-002-A	57	Male	Post-traumatic OA	Left	24	96.5	77.5	10.0
KA-004-B	72	Male	SLAC wrist	Right	24	85.0	80.0	8.4
KA-005-C	62	Male	SNAC wrist	Left	12	100.0	99.2	10.0
KA-006-D	72	Male	SLAC wrist	Right	24	79.0	74.2	8.8
KA-007-E	72	Male	Severe primary OA	Right	12	62.5	64.2	8.0
Mean ± SD	67.0 ± 7.1	—	—	—	19.2 ± 6.6	84.6 ± 15.0	79.0 ± 12.8	9.0 ± 0.9

Demographic, diagnostic, and preoperative clinical characteristics of five consecutive patients who underwent bipolar dual-center total wrist arthroplasty between January and September 2024. The two patients with 12-month follow-up (KA-005-C and KA-007-E) were the most recently operated on, and the three patients with 24-month follow-up underwent surgery earliest in the enrollment period

* Pain was assessed using the pain subscale of the Patient-Rated Wrist Evaluation (PRWE), normalized to a 0–10 scale by dividing the summed subscale score (range, 0–50) by 5

All patients were presented with advanced wrist dysfunction and severe preoperative pain. The mean baseline PRWE score was 84.6 ± 15.0, mean DASH score was 79.0 ± 12.8, and mean PRWE-derived pain score was 9.0 ± 0.9 preoperatively. The mean follow-up was 19.2 ± 6.6 months (range, 12–24 months), with extended 24-month follow-up data available for three patients (KA-002-A, KA-004-B, and KA-006-D).

### Operative characteristics and safety profile

All procedures were performed as planned without intraoperative abandonment or component exchange. A complete summary of the intraoperative events, postoperative adverse events, secondary procedures, and implant status is provided in Table 2. One intraoperative capitate fracture occurred in patient KA-007-E, who was judged to have poor capitate bone quality. The defect was filled with cement, and the carpal component was stabilized without component exchange. No subsequent implant migration or revisions were observed.

**Table 2:** Operative characteristics, safety profile, and implant status

Patient ID	Intraoperative event	Postoperative adverse event	Secondary procedure	Implant status*
KA-002-A	None	None	None	Stable
KA-004-B	None	None	CTR at 8 months	Stable
KA-005-C	None	Traumatic fall onto operated wrist (1–3 months)	CTR at 6 months	Stable
KA-006-D	None	None	CTR at 4 months	Stable
KA-007-E	Capitate fracture during preparation; cement-stabilized; no component exchange	None	None	Stable
Total	1/5	1/5	3/5 CTR; 0/5 revision arthroplasty	5/5 (100%)

The documented intraoperative events, postoperative adverse events, secondary procedures, and final implant status for the five patients. No patient required revision arthroplasty, implant removal, or conversion to wrist arthrodesis after surgery. The intraoperative capitate fracture in patient KA-007-E was managed with cement augmentation. The traumatic fall sustained by patient KA-005-C between the 1- and 3-month postoperative visits did not result in radiographic loss of implant alignment or fixation. All three secondary carpal tunnel releases were performed for symptomatic median neuropathy, with reported improvements

*CTR* carpal tunnel release

* Implant status assessed using serial radiographs at the latest available follow-up. "Stable" indicates the absence of loosening, subsidence, migration, dislocation, or component failure

Three patients with positive preoperative signs of carpal tunnel syndrome underwent secondary carpal tunnel release at 4–8 months after arthroplasty, with reported symptomatic improvement. All secondary procedures were limited to soft tissue. No patient required revision arthroplasty, implant removal, or conversion to wrist arthrodesis after surgery.

### Radiographic outcomes

**Fig. 2:**
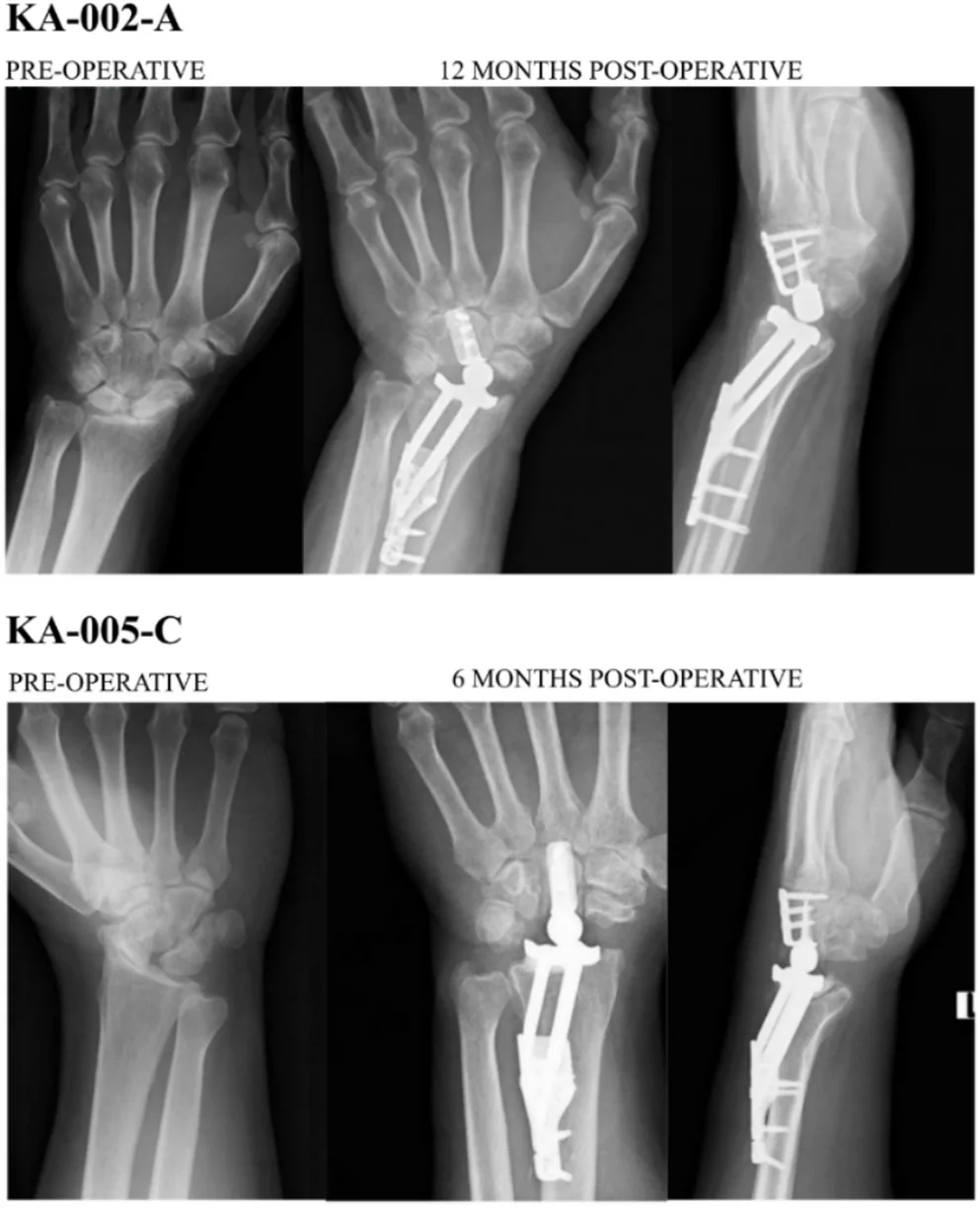
Radiographic evaluation of implant stability. Upper panel: Standard postoperative course (Patient KA-002-A). Preoperative radiograph demonstrating advanced post-traumatic wrist osteoarthritis (left), compared with the 24-month postoperative radiograph (right) showing maintained anatomical alignment and no radiographic signs of implant loosening, subsidence or migration. Lower panel: Mechanical stability after trauma (Patient KA-005-C). Preoperative radiograph of a scaphoid nonunion advanced collapse (SNAC) wrist (left), compared with the postoperative radiograph (right) obtained following a documented traumatic fall onto the operated wrist between the 1- and 3-month follow-up. Implant fixation and alignment were preserved despite adverse events, demonstrating the mechanical resilience of the construct under non-physiological loading. This patient met the predefined wrist-specific responder criteria (ΔPRWE = 30), although residual upper-extremity disability was reflected by his final DASH score

At the latest follow-up, no implant removals or surgical revisions were documented, representing an early component retention rate of 100%. Serial postoperative radiographs revealed no evidence of implant loosening, subsidence, migration, dislocation, or mechanical failure in any patient. One patient (KA-005-C) experienced a documented traumatic fall on the operated wrist between the 1- and 3-month postoperative visits. Despite this adverse event, radiographic evaluation confirmed preserved implant alignment and fixation with no evidence of component failure. Representative radiographs comparing an uncomplicated postoperative course with a trauma case are presented in Fig. 2.

Given the small cohort and short- to intermediate-term follow-up, these findings cannot be extrapolated to reflect definitive long-term survivorship; rather, they serve strictly as a preliminary validation of early mechanical stability under immediate post-operative loading conditions.

### Clinical outcomes

The patient-reported outcomes, wrist range of motion, grip strength, and responder status at the latest available follow-up for each patient are summarized in Table 3.

**Table 3:** Clinical outcomes at latest available follow-up

Patient	FU (mo)	PRWE pre→latest	ΔPRWE	DASH pre→latest	ΔDASH	Pain* pre→latest	ΔPain	Flex/Ext pre→latest	RD/UD pre→latest	Grip pre→latest (kg)	Resp.†
KA-002-A	24	96.5 → 0	96.5	77.5 → 1.7	75.8	10.0 → 0	10.0	20°/20° → 40°/50°	10°/5° → 10°/10°	35.7 → 43.0	Yes
KA-004-B	24	85.0 → 36.0	49.0	80.0 → 38.3	41.7	8.4 → 4.8	3.6	10°/15° → 50°/40°	0°/15° → 15°/15°	12.0 → 22.0	Yes
KA-005-C	12	100.0 → 70.0	30.0	99.2 → 91.7	7.5	10.0 → 7.0	3.0	15°/15° → 10°/20°	0°/10° → 10°/5°	10.1 → 14.1	Yes
KA-006-D	24	79.0 → 0	79.0	74.2 → 0	74.2	8.8 → 0	8.8	10°/0° → 70°/20°	3°/3° → 15°/15°	18.0 → 21.7	Yes
KA-007-E	12	62.5 → 10.0	52.5	64.2 → 15.8	48.4	8.0 → 3.0	5.0	10°/5° → 15°/30°	3°/0° → 5°/5°	13.7 → 25.3	Yes
Mean ± SD	19.2 ± 6.6	84.6 → 23.2	61.4 ± 26.3	79.0 → 29.5	49.5 ± 28.0	9.0 → 3.0	6.1 ± 3.1	13°/11° → 37°/32°	3°/7° → 11°/10°	17.9 → 25.2	5/5

Patient-reported outcomes, wrist range of motion, ipsilateral grip strength, and responder status were compared between the preoperative baseline and the latest available follow-up. Clinical outcomes were analyzed using the latest available follow-up data for each patient (24 months for KA-002-A, KA-004-B, and KA-006-D; 12 months for KA-005-C and KA-007-E). The range of motion values are presented as flexion/extension and radial/ulnar deviation. All five patients met the predefined responder criteria. Notably, all patients achieved ΔPRWE ≥ 30, exceeding the upper bound of the published MCID estimates and supporting the robustness of the responder classification

*DASH* Disabilities of the Arm, Shoulder, and Hand questionnaire,* FU* follow-up,* MCID* minimal clinically important difference,* PRWE* Patient-Rated Wrist Evaluation,* RD* radial deviation, *Resp.* responder,* SD* standard deviation,* UD* ulnar deviation,* Δ* change from baseline

*Pain was assessed using the PRWE pain subscale, normalized to a 0–10 scale (sum of five items divided by 5)

†Responder defined a priori as ΔPRWE ≥ 20 from baseline to latest follow-up (within the published minimal clinically important difference range of 17–24 for individual patient comparisons, and exceeding the lower MCID estimate of 11.5 reported for distal radius fractures) combined with the absence of radiographic signs of implant loosening at the latest available follow-up

### Patient-reported outcomes

**Fig. 3:**
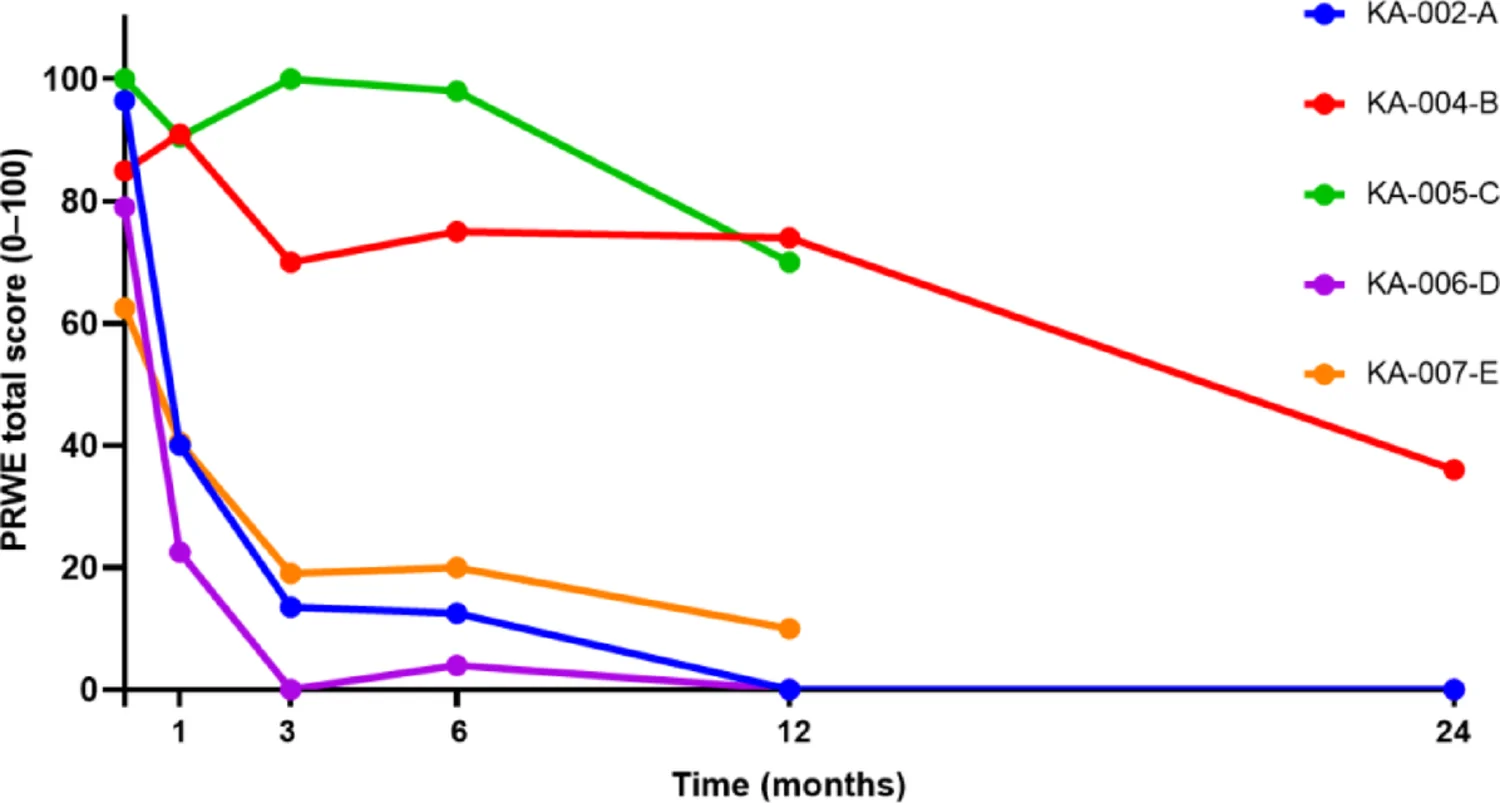
Longitudinal trajectories of PRWE total score. Individual patient trajectories of the Patient-Rated Wrist Evaluation (PRWE) total score from baseline to the latest available follow-up. Each colored line represents one patient (KA-002-A, blue; KA-004-B, red; KA-005-C, green; KA-006-D, purple; and KA-007-E, orange). The shaded region marks the period in which patient KA-004-B exhibited a prolonged functional plateau (3–12 months) prior to a substantial improvement between 12 and 24 months. The dashed horizontal line indicates the responder threshold, defined a priori as ΔPRWE ≥ 20 from the baseline. The annotated event for patient KA-005-C corresponds to his documented traumatic fall onto the operated wrist between the 1- and 3-month visits, after which his PRWE score transiently returned to baseline before partial recovery. The number of patients (n) contributing data at each time point is indicated below the x-axis. Lower PRWE scores indicate better wrist-specific functions. PRWE: Patient-Rated Wrist Evaluation (range 0–100)

**Fig. 4:**
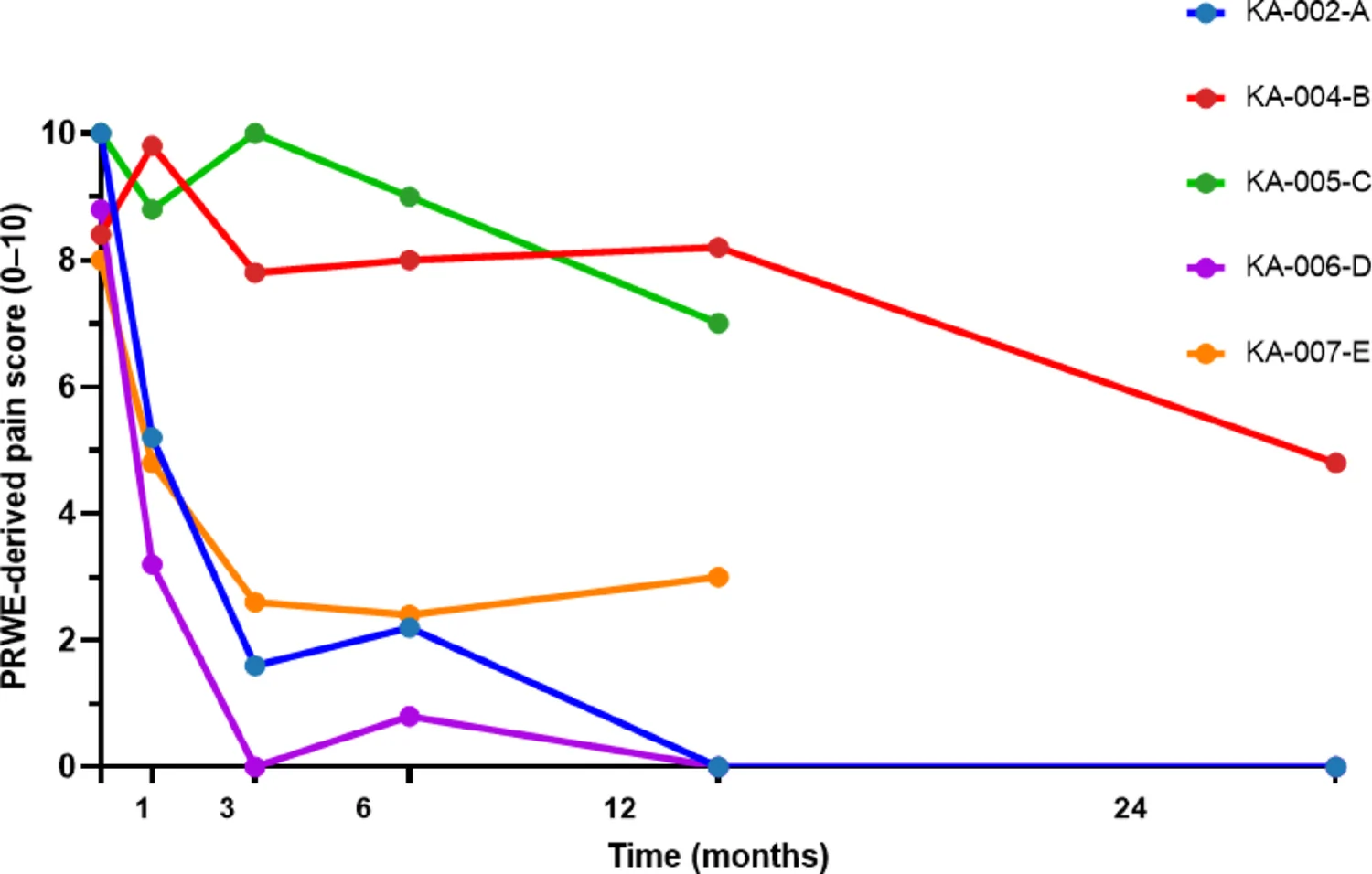
Longitudinal trajectories of PRWE-derived pain score. Individual patient trajectories of pain were assessed using the pain subscale of the Patient-Rated Wrist Evaluation (PRWE) normalized to a 0–10 scale (sum of five items divided by 5). The color coding is identical to that in Fig. 3. Patient KA-004-B exhibited persistent elevated pain through the 12-month visit, followed by a substantial reduction by 24 months. Patient KA-005-C demonstrated a transient return to maximal pain (score 10) at the 3-month visit, corresponding to his documented traumatic fall with subsequent partial recovery. The number of patients (n) contributing data at each time point is indicated below the x-axis. Lower scores indicated less pain

Preoperatively, the PRWE-derived pain scores ranged from 8.0 to 10.0. At the latest available follow-up, the mean pain score improved from 9.0 ± 0.9 to 3.0 ± 3.1, corresponding to a mean reduction of 6.1 points (Table 3 and Fig. 3). Three patients achieved complete pain resolution, with PRWE-pain scores of 0. Longitudinal pain trajectories demonstrated early and sustained pain reduction in most patients, with one patient (KA-004-B) showing persistent pain through 12 months, followed by a substantial improvement by 24 months (Fig. 4).

The mean PRWE total score improved from 84.6 ± 15.0 preoperatively to 23.2 ± 30.0 at the latest follow-up (Table 3). Three patients achieved final PRWE scores ≤ 10, indicating minimal wrist-specific disability. The mean DASH score improved from 79.0 ± 12.8 to 29.5 ± 38.0 postoperatively. The longitudinal PRWE total trajectories are shown in Fig. 3. A notable temporal pattern was observed in patient KA-004-B: functional scores showed a prolonged plateau between 3 and 12 months (PRWE 70–75), with substantial improvement becoming apparent only between 12 and 24 months, when this patient ultimately demonstrated marked improvement across all metrics (PRWE 36, DASH 38.3, and pain 4.8). Discordance between wrist-specific and global upper-extremity outcomes was observed in patient KA-005-C, who met the wrist-specific responder threshold despite persistent limitations reflected by the DASH. This borderline trajectory is discussed further in the Discussion section of this paper.

### Range of motion

The mean wrist flexion improved from 13.0° ± 4.5° preoperatively to 37.0° ± 24.9° at the latest follow-up. The mean extension improved from 11.0° ± 8.2° to 32.0° ± 13.0°. The mean radial deviation improved from 3.2° ± 4.1° to 11.0° ± 4.2°, and the mean ulnar deviation improved from 6.6° ± 5.9° to 10.0° ± 5.0° (Table 3). Patient KA-005-C was the only patient who demonstrated a decrease in any range-of-motion parameter (active wrist flexion 15° to 10° and ulnar deviation 10° to 5°), coinciding with his documented traumatic fall. No mechanical block, implant dislocation, or radiographic loss of alignment was identified in any patient during follow-up.

### Grip strength

The mean ipsilateral grip strength increased from 17.9 ± 10.4 kg preoperatively to 25.2 ± 10.7 kg at the latest available grip strength assessment, corresponding to a mean gain of 7.3 kg (Table 3). Individual grip strength gains ranged from +3.7 kg (KA-006-D) to +11.7 kg (KA-007-E), with all five patients exceeding the baseline at the latest follow-up. Patient KA-006-D, who showed transient grip strength reduction during the first 6 postoperative months, demonstrated a robust recovery by 24 months (21.7 kg), exceeding baseline values.

### Responder analysis

Using the responder criteria defined in the Methods section (ΔPRWE ≥ 20 combined with radiographic stability), all five patients (100%) met the criteria at their latest available follow-up (Table 3). All patients remained radiographically stable at the latest available follow-up, with no evidence of loosening, migration, dislocation, or implant-related mechanical failures.

## Discussion

This pilot study reports the first clinical experience with a novel bipolar dual-center total wrist arthroplasty (TWA) system designed to preserve the radiocarpal and midcarpal centers of rotation. The principal findings of this study are as follows: First, prosthesis implantation was technically feasible in all cases. Second, short-term implant survival was 100%, with radiographic stability preserved in all cases, including after a postoperative traumatic fall and intraoperative capitate fracture. Third, the clinical outcomes were favorable across the cohort, with all five patients meeting the predefined responder criteria, which combined wrist-specific functional improvement with radiographic mechanical stability.

The most notable clinical finding was the consistent achievement of a responder status across all five patients. Heterogeneity in the trajectory of clinical response was observed, as illustrated by the longitudinal patient-reported outcome trajectories (Figs. 2 and 3). Patient KA-004-B demonstrated a delayed recovery pattern, characterized by a prolonged functional plateau between 3 and 12 months, followed by substantial gains between 12 and 24 months (ΔPRWE = 49, ΔDASH = 41.7, Δpain = 3.6). Multiple patient-specific factors may have contributed to this delayed trajectory, including advanced age (this patient was the oldest in the cohort) and documented variability in adherence to the recommended occupational therapy schedule, although these observations are post-hoc, and the underlying mechanisms remain incompletely understood. Patient KA-005-C achieved a responder status but with a more modest improvement. A documented traumatic fall onto the operated wrist between the 1- and 3-month postoperative visits coincided with a transient return of the PRWE score to baseline levels at 3 months (PRWE 100), with partial recovery thereafter (PRWE 70 at 12 months). These factors contributed to the persistence of upper-extremity limitations, as reflected by the DASH score. These observations underscore that while the implant provides reliable mechanical performance, individual patient factors, including age, rehabilitation engagement, and intercurrent adverse events, strongly influence the pace and magnitude of functional recovery.

The interpretation of clinical success in this pilot series relied primarily on wrist-specific metrics (PRWE) rather than global upper extremity scores (DASH). This hierarchical approach is supported by comparative psychometric studies superior responsiveness and construct validity for wrist arthroplasty and distal radius pathologies [17–20]. In our cohort, this distinction was clinically relevant; patient KA-005-C demonstrated a discrepancy between a successful wrist-specific outcome and a poorer global score, likely attributable, in part, to his documented extra-articular trauma and persistent upper-extremity symptoms. Furthermore, to ensure the robustness of our responder definition, we used a ΔPRWE threshold of ≥ 20, which falls within the published MCID range of 17–24 for individual patient comparisons reported in the most recent meta-analysis of PRWE measurement demonstrating that, while the DASH is well-suited for multi-joint disorders, the PRWE exhibits properties [20], and is approximately 1.7-fold greater than the lower MCID estimate of 11.5 reported by Walenkamp et al. for distal radius fractures [16]. This conservative threshold reduces the likelihood that classified responders represent marginal changes or measurement errors. Importantly, all five patients exceeded ΔPRWE = 30, surpassing the upper bound of the published MCID estimates [16, 20, 21]. Therefore, the responder classification was robust regardless of the choice of threshold across the entire reported MCID range, reducing the possibility that the favorable response rate reflected the selection of a permissive cutoff.

A key observation supporting the mechanical concept of dual-center design is the absence of implant-related mechanical failure in all five patients in this study. No cases of loosening, subsidence, migration, or dislocation were observed on radiography. This remained true even in the setting of adverse events, including a documented postoperative traumatic fall in patient KA-005-C and an intraoperative capitate fracture in patient KA-007-E (Fig. 2). These findings suggest that the bipolar dual-center construct may tolerate non-physiological loading without compromising fixation, at least in the short- to intermediate-term. Furthermore, because the implant does not rely on biological ingrowth, long-term stability depends entirely on the integrity of the cement–bone interface, implant–cement interface, and mechanical construct. Therefore, although early radiographic stability is encouraging, it does not reliably predict long-term implant survival. The risk of late aseptic loosening under repetitive cyclic loading remains a critical question that can only be answered through extended longitudinal surveillance of larger cohorts.

The active range of motion at the latest follow-up was modest (mean flexion, 37°; mean extension, 32°). These values likely reflect the natural processes of postoperative soft-tissue scarring and capsular tightness that develop following extensive dorsal wrist exposure, as well as individual variability in the healing response and adherence to the prescribed rehabilitation schedule. Grip strength improvement was also variable, with one patient (KA-006-D) showing a transient reduction during the first 6 postoperative months (from baseline, 18 kg to a nadir of 9 kg at 6 months), followed by recovery to above-baseline values by 24 months (21.7 kg). This nonlinear recovery pattern further supports the value of extended follow-up when interpreting functional outcomes after motion-preserving wrist arthroplasty.

Although the diversity of surgical indications in this cohort, encompassing post-traumatic osteoarthritis, SLAC wrist, SNAC wrist, and primary osteoarthritis, might initially appear to be a limitation, it actually serves as a strength. This variety mirrors real-world clinical practice and offers preliminary insights into the effectiveness of implants across a spectrum of clinical scenarios. The achievement of responder status in all five patients across these heterogeneous indications supports the potential applicability of the dual-center concept in a diverse patient population. Concurrently, the observation that recovery trajectories varied substantially according to age, rehabilitation adherence, and intercurrent events suggests that patient counseling regarding expected recovery timelines remains an important component of patient selection. The notable incidence of secondary carpal tunnel release (60%) in this series highlights the profound clinical overlay of median neuropathy in patients with end-stage wrist arthropathy, rather than a device-related adverse event. Pre-existing neuropathic conditions may require staged management. Based on these observations, we suggest that future clinical protocols incorporate routine preoperative electrodiagnostic screening, and we recommend considering concurrent prophylactic carpal tunnel release during the index arthroplasty to optimize global upper-extremity functional recovery.

An important consideration for future applications of this dual-center design is its performance in patients with compromised bone quality, such as those with rheumatoid arthritis (RA). Although RA is an intended indication for this device, patients with profound bone loss were excluded from this pilot cohort. The implant was designed to achieve robust primary stability through a specific geometric construct combined with targeted cementation. However, the long-term durability of distal fixation in the capitate remains unknown among patients with severe osteopenia or inflammatory arthropathy. Future clinical investigations with longer follow-up periods are required to evaluate the mechanical survivorship of this fixation strategy, specifically within the RA population.

This study has several limitations inherent to its design as a first-in-human pilot study. First, the small sample size (*n* = 5) and single-center setting limit the generalizability of our findings and preclude rigorous statistical analysis. Second, the follow-up duration was variable across the cohort, with extended 24-month data available for three of the five patients and 12-month data for the remaining two, which does not allow for the assessment of long-term implant survival or late complications such as aseptic loosening. Third, the lack of a control group receiving standard single-center TWA or wrist fusion prevented a definitive comparison of the efficacy. Fourth, our cohort consisted exclusively of male patients, and the surgical indications were heterogeneous, which may have introduced a selection bias. Fifth, while a standardized rehabilitation pathway was applied, individual variability in patient adherence and healing response likely contributed to the modest active range of motion observed at follow-up. Sixth, we did not utilize a traditional Visual Analog Scale (VAS, 0–10) for pain at rest and during activity, relying instead on the validated PRWE pain subscale. Future research should address these limitations through larger multicenter studies with longer follow-up periods and further refinement of the standardized rehabilitation protocols. Finally, given the pilot nature of this first-in-human study, formal blinded radiographic adjudication by an independent reader was not performed. Although strict objective criteria for radiolucency (≥ 2 mm) were uniformly applied by the operating surgeon to minimize subjectivity, future multicenter trials must incorporate independent radiographic evaluation to definitively validate these early structural observations.

## Conclusions

In this first-in-human pilot case series, the WristArt^™^ bipolar dual-center total wrist arthroplasty demonstrated technical feasibility and robust short-term mechanical stability, with no evidence of implant loosening. The clinical results were promising, with 100% of the patients achieving meaningful pain relief and functional improvement. While these initial clinical trends are favorable, they must be interpreted with extreme caution, given the profound design limitations, including the very small sample size (*n* = 5), short-to-intermediate follow-up, and absence of a comparative control group inherent to a first-in-human pilot design. Larger, longer-term, and comparative studies are warranted to confirm safety, durability, and clinical efficacy.

## Data Availability

The de-identified datasets generated and analysed during the current study are not publicly available due to patient privacy considerations but are available from the corresponding author on reasonable request, subject to institutional and ethical approval.
